# Comparison of recovery profiles in patients with Parkinson's disease for 2 types of neuromuscular blockade reversal agent following deep brain stimulator implantation

**DOI:** 10.1097/MD.0000000000018406

**Published:** 2019-12-27

**Authors:** Yong-Seok Park, Jaewon Kim, Sung-Hoon Kim, Young-Jin Moon, Hye-Mee Kwon, Hee-Sun Park, Wook-Jong Kim, Seungil Ha

**Affiliations:** a Department of Anesthesia and Pain Medicine, Asan Medical Center, University of Ulsan College of Medicine, Seoul; b Department of Biomedical Science and Engineering, Gwangju Institute of Science and Technology, Gwangju, Republic of Korea.

**Keywords:** neuromuscular blockade, Parkinson's disease, sugammadex

## Abstract

As an anesthetic reversal agent, there are concerns with cholinesterase inhibitors regarding worsening of Parkinson's disease (PD)-related symptoms. Sugammadex, a relatively new reversal agent, does not inhibit acetylcholinesterase and does not require co-administration of an antimuscarinic agent. The present study compared the recovery profiles of 2 agents initially administered for reversal of neuromuscular blockade in patients with advanced PD who underwent deep brain stimulator implantation.

A total of 121 patients with PD who underwent deep brain stimulator implantation were retrospectively analyzed. Patients were divided into 1 of 2 groups according to the type of neuromuscular blockade reversal agent (pyridostigmine vs sugammadex) initially administered. Recovery profiles reflecting time to extubation, reversal failure at first attempt, and hemodynamic stability, including incidence of hypertension or tachycardia during the emergence period, were compared.

Time to extubation in the sugammadex group was significantly shorter (*P* < .001). In the sugammadex group, reversal failure at first attempt did not occur in any patient, while it occurred in seven (9.7%) patients in the pyridostigmine group (*P* = .064), necessitating an additional dose of pyridostigmine (n = 3) or sugammadex (n = 4). The incidence of hemodynamic instability during anesthetic emergence was significantly lower in the sugammadex group than in the pyridostigmine group (*P* = .019).

Sugammadex yielded a recovery profile superior to that of pyridostigmine during the anesthesia emergence period in advanced PD patients. Sugammadex is also likely to be associated with fewer adverse effects than traditional reversal agents, which in turn would also improve overall postoperative management in this patient population.

## 1 Introduction

Parkinson's disease (PD) is a common neurodegenerative disease with a prevalence of 3% in the elderly population.^[1]^ Anesthetic management for patients with PD has been addressed for concerns regarding interactions between anesthetics and anti-PD medications or PD-related symptoms.[^2^,^3^,^4^] In addition, PD patients are known to exhibit abnormal pharmacodynamics of various drugs due to degenerative changes in the neural system. For example, remifentanil requirement for the inhibition of responses to tracheal intubation and surgical incision are markedly reduced in PD patients.^[5]^ As a reversal agent for neuromuscular blockade, there are concerns with cholinesterase inhibitors given the theoretical possibility that they could worsen PD-related symptoms, particularly those involving movement. Furthermore, co-administration of antimuscarinic agents should be exercised with caution because PD patients with cholinergic deficits are particularly sensitive to the effects of anticholinergic drugs.^[6]^

An ideal agent for neurosurgical anesthesia would provide a quick recovery time, enabling early assessment of the patients’ neurological status and rapid recognition of potential postoperative complications.^[7]^ In this regard, sugammadex, a relatively new reversal agent, does not inhibit acetylcholinesterase; therefore, cholinergic effects are not produced and co-administration of an antimuscarinic agent is not required. Patients who were administered sugammadex have been observed to recover with a clearer level of consciousness, compared to those who received neostigmine.[^8^,^9^] Sugammadex also ensures full neuromuscular recovery by encapsulating steroidal neuromuscular blocking agents. When sugammadex was used for reversal, rapid recovery of neuromuscular function was found in patients with various neuromuscular disorders including myasthenia gravis^[10]^ or Becker myotonia congenita.^[11]^

In the present study, we compared the recovery profiles of 2 agents initially used to reverse neuromuscular blockade in PD patients who underwent deep brain stimulator implantation. We hypothesized that sugammadex could reduce extubation time, as well as the occurrence of adverse events during emergence from anesthesia, facilitating early recovery after surgery, even in the absence of neuromuscular monitoring in a routine clinical anesthesia setting. We also sought to explain perioperative recovery profiles in relation to PD related symptoms and postoperative outcomes.

## 2 Methods

Ethics approval for this study was provided by the Asan Medical Center Institutional Review Board, Seoul, Korea. Given the retrospective nature of the present study and the use of anonymized patient data, requirements for informed consent were waived. A total of 121 patients with advanced PD who underwent deep brain stimulator implantation surgery under general anesthesia using rocuronium as the intraoperative neuromuscular blockade agent, without the use of an intraoperative neuromuscular monitoring device between November 2014 and March 2016, were analyzed. Perioperative data were collected through a review of the institutional electronic medical record system.

According to standard anesthesia care at the authors’ institution, anesthesia was induced using propofol 2 mg/kg, with bolus rocuronium 0.6 mg/kg administered to facilitate endotracheal intubation. After intubation, the patients were ventilated with 50% N_2_O in oxygen with a 1.0 to 1.5 vol% of sevoflurane, and end-tidal carbon-dioxide maintained at a partial pressure between 30 and 35 mmHg. Esophageal temperature was maintained at >35.5°C using a warming blanket. Patients were divided into one of two groups according to the type of initially chosen neuromuscular blockade agent—pyridostigmine or sugammadex—at the discretion of the attending anesthesiologist. The dose of sugammadex was 200 mg and the dose of pyridostigmine and glycopyrrolate were 15 mg and 0.4 mg, respectively. All data, including patients’ characteristics and outcomes, were collected from the electronic medical record system by a single investigator blinded to the study design. The primary outcome was extubation time, which was measured as the time interval from the conclusion of surgery to extubation. Secondary outcomes included reversal failure at first attempt (>30 minutes of extubation time), postoperative adverse events in the post-anesthesia care unit, including respiratory and hemodynamic complications, postoperative residual weakness, postoperative nausea and vomiting, oxygen desaturation (SpO_2_ < 93%), laryngospasm, and hemodynamic stability, such as incidence of hypertension or tachycardia, during the anesthesia emergence period. Hypertension and tachycardia were considered to be present if the values exceeded 20% of the baseline values. Postoperative hospital stay, readmission rate and unforeseen intensive care unit rate were assessed as long-term outcomes.

Statistical analyses were performed using SPSS version 12.0 (SPSS Inc, Chicago, IL) and R version 3.4.2 (R Foundation for Statistical Computing, Vienna, Austria). Categorical data, including sex, diabetes mellitus, hypertension, and the incidence of postoperative adverse events between the groups were compared using the Chi-squared or Fisher exact tests. Other parametric data, including age, height, anesthesia time, and extubation time, between 2 groups were compared using a two-tailed *t* test or the Mann–Whitney *U* test. Based on our in-house clinical cohort (n = 20), expected standard deviation of extubation time for Parkinson's disease patients was 11 minutes. Assuming that meaningful difference of extuabtion time for both groups was 8 minutes with power 0.9 and alpha 0.05, the minimum size of the 2 groups was 41 per group. Kaplan–Meier curves for extubation time were constructed, and the log-rank test was used to compare extubation time between the groups.^[12]^ *P* < .05 was considered to be statistically significant.

## 3 Results

**Figure 1:**
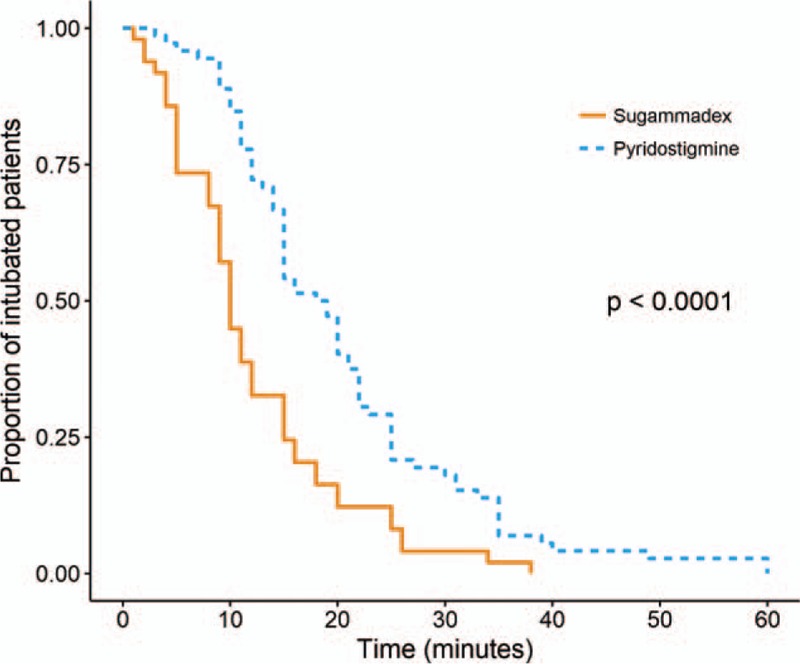
Kaplan-Meier curve for the proportion of patients still intubated after the completion of surgical dressing. Median extubation time in the pyridostigmine (blue line) and sugammadex (orange line) groups were 21 min (interquartile range, 17–25 min), and 11 min (interquartile range, 9–13 min), respectively (*P* < 0.001 [log-rank test]).

A total of 121 patients were evaluated, with 49 included in sugammadex group and 72 in pyridostigmine group. There were essentially no differences in patient demographic data between the groups; however, patients in sugammadex group were significantly older than those in pyridostigmine group (*P* = .010) (Table 1). Additional dose of rocuronium was administered to 61 patients (61.1%) in pyridostigmine group and to 31 (63.3%) in sugammadex group (*P* = .961). The incidences of 2 or more additional administration of rocuronium were 24 (33.3%) in pyridostigmine group and 25 (51.0%) in sugammadex group (*P* = .079). Time to extubation in the sugammadex group was significantly shorter than in the pyridostigmine group (12.0 ± 8.1 min vs 20.4 ± 11.5 min, respectively; *P* < .001) (Table 2). The proportion of patients still intubated after the completion of surgical dressing is presented in Figure 1. No patient in the sugammadex group failed neuromuscular blockade reversal at first attempt, while seven (9.7%) patients in pyridostigmine group failed at first attempt (*P* = .064), and required an additional dose of pyridostigmine (n = 3) or sugammadex (n = 4). The total amount of administered rocuronium was significantly higher in the sugammadex group (69.0 ± 25.6 mg) than in the pyridostigmine group (59.7 ± 13.7 mg) (*P* = .023).

**Table 1:**
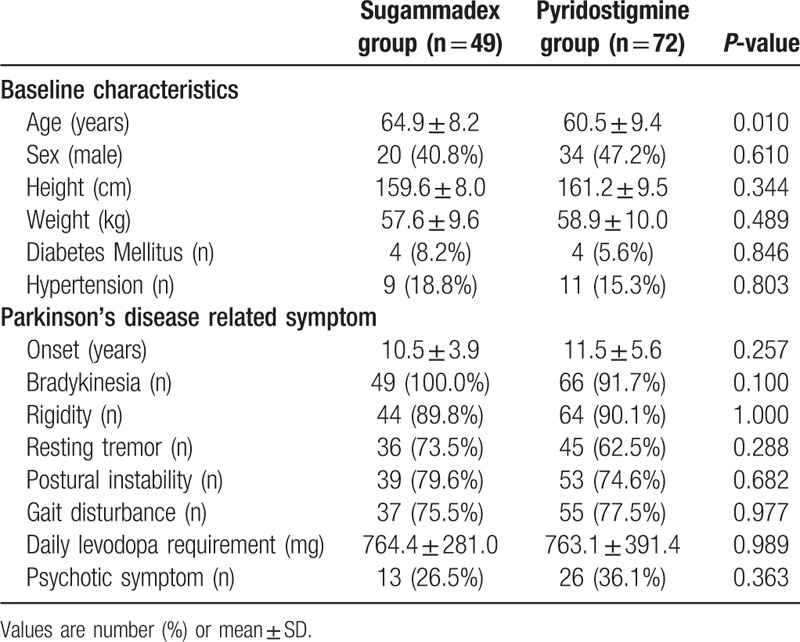
Patient demographic data.

**Table 2:**
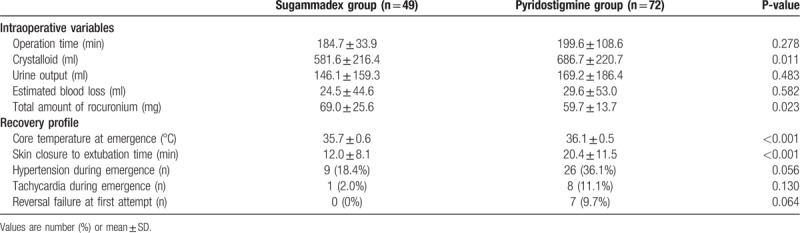
Intraoperative variables and recovery profile.

**Figure 2:**
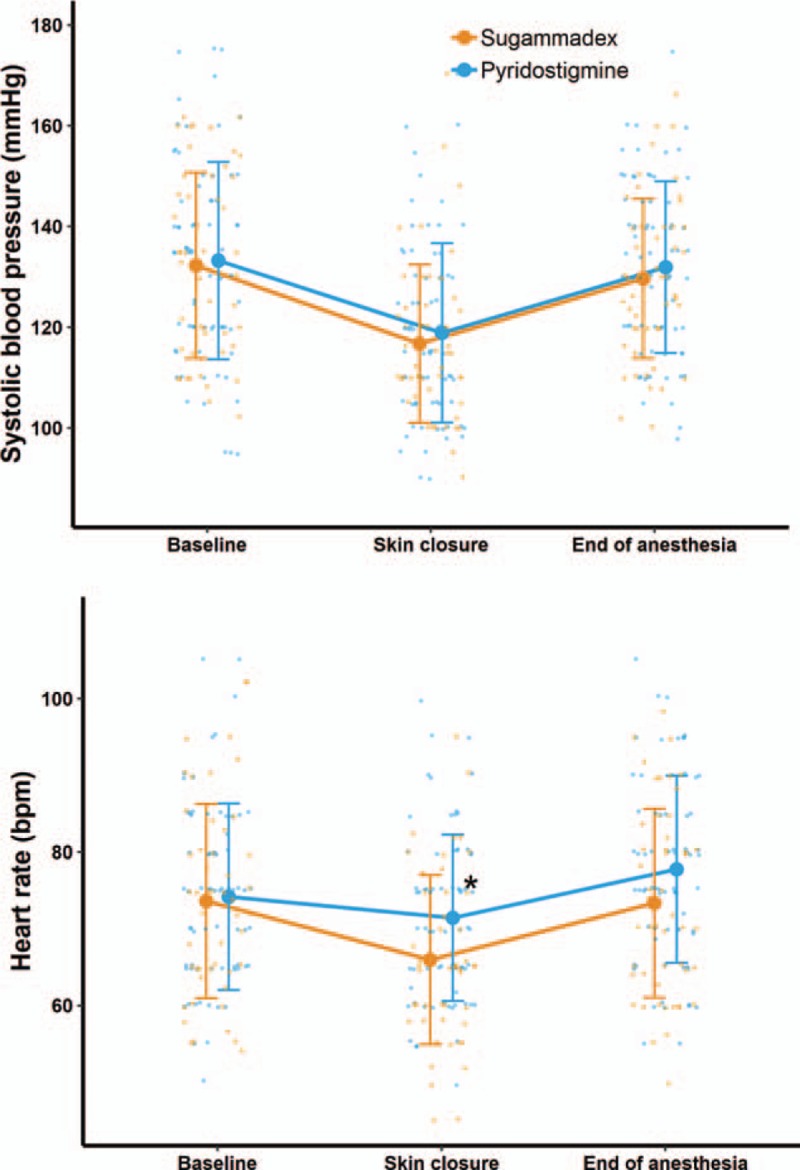
Hemodynamic changes at baseline (before induction of anesthesia), at skin closure (administration of neuromuscular blockade reversal agent), and at the end of anesthesia (completion of extubation) in both groups. ^∗^ *P* < 0.05.

Adverse events during anesthesia emergence were comparable between the groups, except for hemodynamic adverse events. The overall incidence of hemodynamic events, including hypertension or tachycardia, during emergence were significantly lower in the sugammadex group (n = 9 [18.4%]) than those in the pyridostigmine group (n = 29 [40.3%]) (*P* = .019). However, the individual incidence of hypertension or tachycardia during emergence was not significantly different between the 2 groups (hypertension, 9/49 [18.4%] vs 26/72 [36.1%], *P* = .056; tachycardia, 1/49 [2.0%] vs 8/72 [11.1%], *P* = .130) (Table 2). Intraoperative hemodynamic features were similar between the 2 groups, except for heart rate at the time of skin closure (sugammadex, 66.0 ± 11.0 beats/min vs pyridostigmine, 71.4 ± 10.8 beats/min; *P* = .008) in which the reversal agents were administered (Fig. 2). There was one case of postoperative nausea and vomiting that required rescue antiemetics in the pyridostigmine group and none in sugammadex group; this difference, however, was not statistically significant. The requirement for postoperative analgesics was similar between the 2 groups (sugammadex, n = 4; pyridostigmine, n = 6). There were no desaturation events (i.e., SpO_2_ < 93%) in the sugammadex group; however, there was one such case in the pyridostigmine group. No patient in this study experienced laryngospasm. The core temperature at emergence from anesthesia was significantly lower in the sugammadex group (35.7 ± 0.6°C vs 36.1 ± 0.5°C; *P* < .001). There were no significant differences in long-term outcomes between 2 groups.

## 4 Discussion

Results of the current study demonstrated that sugammadex provided a recovery profile superior to pyridostigmine during the anesthesia emergence period in PD patients undergoing deep brain stimulator implantation. More specifically, time to extubation in the sugammadex group was significantly shorter than in the pyridostigmine group, and no patient in the sugammadex group failed neuromuscular blockade reversal at first attempt. Sugammadex is also expected to have fewer adverse effects, such as hypertension or tachycardia, than traditional reversal agents in patients with PD. From a clinical perspective, the results of this study may be helpful, and are anticipated to improve outcomes in patients with advanced PD undergoing general anesthesia.

Since its introduction, sugammadex has demonstrated promising efficacy and safety, perhaps ringing in a new era of patient safety in clinical anesthesia.[^13^,^14^] The effect of sugammadex in shortening extubation time has been reported in many previous studies, and in settings using appropriate neuromuscular monitoring.[^15^,^16^] In a randomized controlled trial that compared sugammadex and neostigmine in patients undergoing laparoscopic surgery, the recovery from neuromuscular blockade was 3.4 times faster in patients who were administered sugammadex than neostigmine.^[17]^ Sugammadex was also reported to reduce total anesthesia time, the time from end of surgery to end of anesthesia, and hospital stay in donors undergoing living-donor hepatectomy.^[18]^ Similarly, the shortened extubation time and recovery time in the sugammadex group in the current study may enable higher operating room turnover, which in turn can lead to improved patient prognosis in terms of overall safety. Although proportion of two or more additional dose of rocuronium did not reach statistical difference, it implies more patients in sugammadex group received rocuronium closer to the time of extubation. This result reinforce our conclusion that sugammadex reverses neuromuscular blockade effectively regardless of last time or dose of rocuronium in Parkinson's disease patients undergoing DBS implantation surgery. As enhanced recovery after surgery (ERAS) has attracted increasing attention, enhanced recovery protocols for perioperative care have been proven to reduce various complications after surgery, improve overall outcomes, and shorten the length of hospital stay, resulting in improved resource management.^[19]^ Based on the results of the present study, the use of sugammadex as a reversal agent for patients with PD undergoing general anesthesia is likely to improve outcomes.

Regarding adverse events, although not statistically significant, there were seven cases of failure of reversal at first attempt (incomplete reversal) in the pyridostigmine group, which can be a detrimental complication,[^20^,^21^] whereas no such failures were recorded in the sugammadex group. In a prospective observational study conducted at 5 teaching hospitals in Japan, the incidence of residual neuromuscular weakness, defined by the train-of-four (TOF) < 0.9 or < 1.0, were lower in the sugammadex group than in the neostigmine group (TOF < 0.9, 4.3% vs 23.9%, *P* < .001; TOF <1.0, 46.2% vs 67.0%, *P* = .003), although the authors emphasized that a risk for postoperative neuromuscular weakness remained in the sugammadex group.^[22]^ This is consistent with the results of our study, in that the use of sugammadex reduced the failure rate of neuromuscular blockade reversal. Hemodynamic values during emergence from anesthetic were found to be better in the sugammadex group, and the incidence of hemodynamic disturbances, including hypertension and tachycardia, were lower. Similar results were also reported in a previous study involving patients with American Society of Anesthesiologists ASA class I or II undergoing laryngeal microsurgery.^[23]^ Glycopyrrolate injected along with pyridostigmine to block its cholinergic adverse effects may explain the significantly higher incidence of tachycardia in the pyridostigmine group.^[24]^ While sugammadex is likely to be associated with superior recovery profile and fewer adverse effects than pyridostigmine during the anesthesia emergence period, there were no significant differences in long-term outcomes between 2 groups. Although clinical studies assessing patient outcomes following post-anesthesia care unit discharge are scarce, recent study on colorectal surgery population also showed no differences in total length of hospital stay, length of postoperative hospital stay, readmission rates, or postoperative pulmonary complication rates between 2 groups.^[25]^

The choice of reversal agent for neuromuscular blockade was entirely based on the clinical judgment of the attending anesthesiologist. Therefore, it is possible that sugammadex was preferred by the clinician for patients who were likely to experience delayed awakening from anesthesia or residual neuromuscular weakness considering patient status at the time. For example, if an older patient was administered more rocuronium than the usual amount, and his body temperature is low, the attending anesthesiologist would prefer sugammadex as a reversal agent. This may, in part, explain the results of the current study, given that the patients in the sugammadex group were older, were administered more rocuronium, and had lower core temperature at emergence.

This study, however, had a few limitations, the first of which was its retrospective design. We plan to conduct a randomized controlled trial to investigate this topic to further assess its implications for an ERAS protocol for patients with PD in the near future. Second limitation was that a neuromuscular monitoring protocol, such as TOF, was not used because it was not our routine clinical monitoring in this type of surgery. Therefore, quantification of the extent of neuromuscular blockade throughout the operation was not possible and, thus, the degree of neuromuscular blockade reversal by sugammadex or pyridostigmine was determined based on clinical judgment. Nevertheless, the results of current study further demonstrated that the efficacy of sugammadex in patients with advanced PD remains firm in the absence of proper neuromuscular monitoring.^[23]^ Although the results of our study demonstrated the reversal effect of sugammadex in the absence of neuromuscular monitoring, further studies to confirm the degree of neuromuscular blockade in this patient population are warranted.

In conclusion, sugammadex shortened extubation time and reduced the frequency of postoperative hemodynamic events in patients with PD. The results of the current study suggest that sugammadex can be used in deep brain stimulation surgery under general anesthesia in patients with advanced PD and, thus, improve overall postoperative management.

## Author contributions

**Conceptualization:** Sung-Hoon Kim, Yong-Seok Park.

**Data curation:** Sung-Hoon Kim, Yong-Seok Park, Jae-Won Kim, Seungil Ha.

**Formal analysis:** Sung-Hoon Kim, Yong-Seok Park, Jaewon Kim.

**Investigation:** Yong-Seok Park, Young-Jin Moon, Wook-Jong Kim.

**Methodology:** Yong-Seok Park, Jaewon Kim, Sung-Hoon Kim, Young-Jin Moon, Wook-Jong Kim

**Supervision:** Sung-Hoon Kim, Young-Jin Moon, Wook-Jong Kim, Seungil Ha.

**Validation:** Hee-Sun Park, Hye-Mee Kwon, Wook-Jong Kim.

**Writing – original draft:** Yong-Seok Park, Jaewon Kim, Sung-Hoon Kim.

**Writing – review & editing:** Sung-Hoon Kim, Young-Jin Moon, Hye-Mee Kwon, Hee-Sun Park, Wook-Jong Kim, Seungil Ha, Yong-Seok Park, Jae-Won Kim.
